# Questioning Established Theories and Treatment Methods Related to Iron and Other Metal Metabolic Changes, Affecting All Major Diseases and Billions of Patients

**DOI:** 10.3390/ijms23031364

**Published:** 2022-01-25

**Authors:** George J. Kontoghiorghes

**Affiliations:** Postgraduate Research Institute of Science, Technology, Environment and Medicine, 3 Ammochostou Street, Limassol 3021, Cyprus; kontoghiorghes.g.j@pri.ac.cy; Tel.: +357-26272076; Fax: +357-26272076

**Keywords:** metal metabolism, iron chelation, metal related diseases, treatment, metal toxicity, drugs in iron diseases

The medical and scientific literature is dominated by highly cited historical theories and findings. It is important to question these theories and report findings that are deemed to be less significant, as this could introduce new perspectives in theoretical models, which will aim to better understand disease processes and their treatments. The gaps and limitations in theoretical models are usually identified as molecular basic science models are translated into biological models and then further into clinical ones, especially when a wide range of treatment options exist. 

There are many examples in various areas of medicine, including diseases related to iron metabolism, where the translation of basic science information, which is potentially insufficient, could lead to errors when operating disease models and suboptimal treatment for patients. Billions of people are affected by iron-related diseases worldwide, and most of these involve iron deficiency and iron overload, which are treated using iron chelator complexes and chelators that eliminate iron, respectively [1,2,3,4]. Most of the other common diseases affecting humans, such as cancer, neurodegenerative and haematological diseases, infections including COVID-19 and also many others, appear to cause changes in iron metabolic pathways. In most cases, these changes are characterized by abnormal levels in iron diagnostic indices such as the level of haemoglobin, serum ferritin, serum iron, abnormal focal iron deposition in specific cells or tissues, etc. Despite the fact that iron may not be directly implicated in many of these diseases, interventional therapies that restore or improve iron related imbalances may facilitate the treatment of or even improve the prognosis for patients with such diseases [5]. 

In general, the control of iron, which is involved in many iron containing enzymes and metabolic pathways in all organisms including humans, may play a central role in therapeutic protocols involving both common and rare diseases [5]. Similar roles and treatment approaches are considered in the case of other essential metals such as copper and zinc [6,7,8,9,10,11]. 

Abnormal metal metabolic changes and associated metal interactions can also affect therapeutic interventions and the overall treatment of patients. For example, changes in iron metabolism such as iron overload can also affect the metabolism of many other metals including essential, xenobiotic and diagnostic metals. Most of these metals share and compete for the metal transport properties of transferrin and subsequently interfere with the metabolic, therapeutic or diagnostic effects of other metals [12]. Similarly, the metabolism and therapeutic effects of many drugs, as well as the metabolism and nutritional effects of many nutrients, most of which have metal binding properties, can also be affected [13,14,15,16]. All these interactions and their implications on health and disease have not yet been fully investigated. 

The lack of in-depth investigations and inaccurate reporting, as well as distorted, misleading or poorly promoted findings, may also lead to insufficient information and suboptimal patient treatments. This influence is related in most cases to the level of academic promotion and the number of citations of particular theories and is also generally observed to take place in relation to the marketing activities of private companies involved mainly in the sale of new, very expensive medicines and medical devices [17]. 

There are many examples in the literature questioning established theories in the risk/benefit assessment for the use of different drugs, dietary components and other medicines. In particular, the benefits, including the efficacy and low toxicity of traditional/folk medicines, are questioned in the medical literature despite the fact that millions of people are preferably using them in comparison to synthetic drugs. Similarly, millions of people are using routinely old/generic drugs such as the chelating drug ethylenediaminetetraacetic acid (EDTA) because of the perceived multiple health benefits [18,19].

The existence of several drugs for each disease can be beneficial in cases of adverse reactions from one particular drug. In contrast, the selection of a specific drug that is randomly selected from a group of drugs for a particular disease can also lead, in many cases, to suboptimal treatment for patients. For example, the conflict of opinion in the use of different chelating drugs and metal complexes in different clinical conditions and categories of patient is highlighted in the medical literature [17,20,21,22,23,24,25,26,27]. 

In contrast to conflicts that arise from the selection of drugs, differences of opinion in the biology and metabolism of iron and other metal ions is on a much smaller scale compared to the use of different drugs for diseases related to abnormal iron or other metal metabolism. However, such differences and related discussions are necessary for exploring the role of metal ions and their metabolic pathways in the maintenance of metal homeostasis under normal conditions and also for the treatment of many diseases.

An example of a molecular interaction of physiological and toxicological significance, which has been neglected for more than a half a century, is the ferroxidase and potent antioxidant activity of transferrin [12,28]. Despite the fact that caeruloplasmin is promoted and cited in hundreds of relevant textbooks and papers as the protein required for the oxidation of ferrous to ferric iron before its uptake by transferrin, transferrin has also previously been shown to be able to oxidize ferrous to ferric iron before its chelation and the formation of the transferrin ferric iron complex [29,30,31,32,33,34]. The extent of the participation of either caeruloplasmin or transferrin in the ferroxidase activity under physiological conditions has not yet been fully investigated. In the meantime, similar properties have been shown by the iron chelating drugs deferiprone, deferasirox and deferoxamine, which can rapidly oxidize ferrous to ferric iron at physiological conditions before the formation of the corresponding ferric iron complexes [15,35,36,37]. The ferroxidase and iron complex formation activity of the iron chelating drugs is the cornerstone of their antioxidant activity in the Fenton reaction and has been utilized and shown to be related to the powerful antioxidant effects exerted in many in vitro, in vivo and clinical models of oxidative stress [38]. The therapeutic application of chelating drugs in many diseases associated with free radical pathology, as well as the role of transferrin, is currently under investigation [28,39,40]. One major characteristic of chelators including transferrin and chelating drugs is their pro-oxidant effects under certain conditions [41,42,43]. In particular, the pro-oxidant effects of some selective chelator iron and copper complexes have been used in different experimental models for the development of new anticancer drugs [44,45,46].

The transport of iron and other metal ions in and out of different cells is of physiological, pharmacological and toxicological importance. The factors affecting metal ion transport in and out of cells is the subject of continuous investigations and discussions in health and disease. In particular, changes in the regulatory molecules, mechanisms and homeostatic controls involved in the transport of iron and other metal ions could have serious implications for the health statuses of many categories of patients [4,5,47]. Despite the fact that emphasis in the medical literature is primarily given to molecular biology effect changes, chelation and other molecular effects involving metal ions could also have major implications on the transportation, distribution and deposition of metal ions, as well as on the causes or progression of different diseases [4,5,47,48,49,50,51,52]. In general, natural or synthetic lipophilic metal chelator complexes can increase cellular metal ion intake and increase metal ion absorption, whereas hydrophilic chelators can remove intracellular metal ion and increase metal ion excretion [4,5,44,49]. In particular, the increase of iron absorption by lipophilic iron chelator complexes is important for all those affected by iron deficiency and mostly vegetarian populations [53].

In addition to the effects of the chelation of metals, there are many other unexplored areas in relation to metal ion metabolism and associated diseases. One such example is the impact of the regulatory excretion of natural and xenobiotic metal ions. Despite the fact that the homeostatic controls, mechanisms and route of the regulatory excretion of copper and zinc have been partly determined, no such information is available for iron and many of the diagnostic metal ions, all of which are important for the diagnosis and treatment of different diseases including iron overload [54,55,56,57,58,59]. In particular, the elimination of excess iron in the absence of chelation therapy in post-transplanted thalassaemia patients and some other categories of patients requires further investigation [60,61]. 

A major unexplored area related to many diseases is the identification of the mechanisms and factors involved in the abnormal distribution and deposition of metal ions in different cells and tissues. In particular, the abnormal distribution and deposition of iron has been observed in many diseases with high morbidity and mortality rates, including many cancer, haematological, neurodegenerative and infectious diseases including COVID-19 [4,5]. Although iron may not directly be implicated in the fatal or serious pathophysiological effects in these diseases, iron abnormalities can constitute a major factor in the progress and outcome of the pathological effects of the underlying disease and also their treatment. Within this context and whenever appropriate, iron chelating drugs and other chelators and chelator iron complexes could be introduced to restore iron balance and improve the overall therapeutic effects, including combination therapies with other drugs [4,5]. 

The targeting of specific mechanisms and pathways involving iron and other metal ion abnormalities is important for many diseases. An example is the major role of macrophages in the abnormal distribution and deposition of iron in many malignant, inflammatory and infectious diseases including COVID-19, which in most cases is also characterized by an elevation in serum ferritin and a reduction in serum iron levels [62,63,64,65,66,67]. The mechanisms involved in these changes are not yet fully clarified. Similarly, therapeutic interventions, which may include chelating drugs for the targeting of specific pathways involving macrophage iron and related changes, as well as other relevant targets need further investigation [28,68,69,70]. 

The diagnosis and treatment of different conditions related to iron and other metal metabolic abnormalities are among the major areas of conflict and controversy involving marketing tactics for the promotion of different medical diagnostics and drugs by pharmaceutical and other companies. In general, the marketing activities of private companies affect academic research and the reporting of clinical findings and also, most importantly, the morbidity and mortality rate of billions of patients [4,5,25,53,71,72,73]. For example, in the case of iron deficiency anaemia, hundreds of different chelator and other iron complexes are promoted, especially of new and expensive formulations claiming effective treatments and superiority over other inexpensive, generic formulations [53,74,75]. 

Similar competition takes place in the case of iron chelation therapy for the removal of excess toxic iron in iron overload conditions, where there is conflicting opinion in the selection of the appropriate drug between deferoxamine, deferiprone and deferasirox. However, only deferiprone and the deferiprone/deferoxamine combination have been shown to remove iron from the heart and all other major organs effectively, as well as returning body iron levels to those similar to normal individuals [76,77,78]. Furthermore, in a long term follow up study, it has been shown that the deferiprone/deferoxamine combination can increase the long term survival of thalassaemia patients [79]. It should be noted that the combination of drugs with similar pharmacological activity such as the deferiprone/deferoxamine combination is more effective than either of the monotherapies, but such therapeutic strategies are not promoted by pharmaceutical companies or even by drug regulatory authorities [72]. 

Conflicting theoretical models and research findings involving academic researchers sponsored by pharmaceutical companies are also widely discussed in the medical and other academic literature. In many cases, elite journals are also involved in such promotions by pharmaceutical companies, especially when publishing questionable theoretical models and findings, causing confusion to patients [72]. There are many examples such as the categorization of thalassaemia intermedia in the so called “non-transfusion-dependent-thalassaemia” group, where deferasirox was promoted as a new therapy despite the fact that deferoxamine and deferiprone have been used in this category of patients for over 50 years [72,80,81]. 

Another major issue affecting the safety and survival of patients in diseases related to iron and other metal metabolism, as well as chelation therapies, is the interaction of chelating drugs and chelator metal complexes with other drugs and therapies [13,14,82,83,84,85,86]. In this context, such interactions may be critical and established related treatment protocols involving such interactions have to be modified. In particular, the safety and efficacy of newly approved drugs in many cases is questionable because of the lack of sufficient long term clinical outcome data. For example, the introduction of erythropoietic biologics such as luspatercept (Reblozyl) has been considered for reducing red blood cell transfusions in haematological diseases including thalassaemia, where it has been suggested that a 20% reduction is possible [87,88,89]. However, the use of luspatercept in thalassaemia major is questionable since, even if haematopoiesis can be increased in patients, this can only produce abnormal, non-functional haemoglobin. Similarly, serious concerns remain in other aspects of therapy, such as the safety, efficacy and the cost of luspatercept and also of other biologics, including those used in the treatment of COVID-19, since, in general, the immunogenicity and other toxic side effects of these compounds have not yet been fully investigated [90,91]. 

In conclusion, established theoretical models and principles in science and medicine have to be scrutinized and re-evaluated at all times, with an emphasis on the improvement of knowledge and, where appropriate, the introduction of better treatments for the increased survival and safety of patients. In cases of pharmaceutical products, the influence of private companies including the promotion of their new products through the medical literature may affect the morbidity and mortality rate of many categories of patients. Herein, several examples examining the validity of a number of established theoretical models and drugs associated with diseases related to iron and other metal metabolic diseases have been questioned. It is hoped that further discussions and re-evaluation could lead in the design of better theoretical models in science and medicine related to iron and other metal metabolism and, most importantly, for safer and more effective treatments for patients. 
